# Quantifying the Effect of Macroeconomic and Social Factors on Illegal E-Waste Trade

**DOI:** 10.3390/ijerph13080789

**Published:** 2016-08-05

**Authors:** Loukia Efthymiou, Amaryllis Mavragani, Konstantinos P. Tsagarakis

**Affiliations:** Business and Environmental Technology Economics Lab, Department of Environmental Engineering, School of Engineering, Democritus University of Thrace, Vas. Sofias 12, Xanthi 67100, Greece; luxloukia@hotmail.com (L.E.); amavraga@env.duth.gr (A.M.)

**Keywords:** e-waste, human development index, illegal trade, open markets index, social progress index

## Abstract

As illegal e-waste trade has been significantly growing over the course of the last few years, the consequences on human health and the environment demand immediate action on the part of the global community. Though it is argued that e-waste flows from developed to developing countries, this subject seems to be more complex than that, with a variety of studies suggesting that income per capita is not the only factor affecting the choice of regions that e-waste is illegally shipped to. How is a country’s economic and social development associated with illegal e-waste trade? Is legislation an important factor? This paper aims at quantifying macroeconomic (per capita income and openness of economy) and social (human development and social progress) aspects, based on qualitative data on illegal e-waste trade routes, by examining the percentage differences in scorings in selected indicators for all known and suspected routes. The results show that illegal e-waste trade occurs from economically and socially developed regions to countries with significantly lower levels of overall development, with few exceptions, which could be attributed to the fact that several countries have loose regulations on e-waste trade, thus deeming them attractive for potential illegal activities.

## 1. Introduction

Pollution-based crime, a category of environmental crime, is considered to be the act of illegally trading and disposing of hazardous waste [1]. Illegal e-waste dumping or burning falls under the definition of environmental crime (though white collar [2]), in that the waste of electrical and electronic equipment contains hazardous materials [2] that could harm the environment [3,4] and human health [5,6,7]. Impacts are also caused by informal and improper e-waste management [3,4], especially in developing countries [7], as e-waste carries toxic components—such as cadmium, mercury and lead—that make it extremely dangerous [8].

The main reasons for illegal e-waste trafficking are weak regulatory enforcement, and the fact that it is more costly to locally and legally treat/recycle e-waste than it is for the e-waste to be illegally shipped to specific regions [2,9,10]. In addition, e-waste contains valuable raw materials, such as gold and copper, making the recovery of these substances profitable for developing countries [8]. Several international regulations have been issued in order to deal with waste crime and, consequently, illegal e-waste dumping or burning. The most relevant regulation, the Basel Convention and its Ban Amendment, seeks to provide a framework where transboundary movements of hazardous wastes should be illegal [11]. Though the EU has regulated these transboundary movements by issuing Directive 2002/96/EC [12], illegal e-waste trafficking has not been limited due to the fact that there is no worldwide legislation. Developed regions (e.g., the EU) are not allowed to export e-waste to non-OECD countries for treatment or disposal, due to the lack or weakness of the aforementioned regulatory enforcement [2]. Though about 70% of all e-waste is shipped to China [10,13], in light of recent regulations adopted in the region, it could be the case that West Africa is to receive larger amounts of e-waste in the coming years [9].

Developed countries are the countries that are economically and socially developed, with high industrial activity, while developing countries are those with low levels of industrial, economic, and social development. It has been suggested by previous studies that e-waste flows from developed to developing countries [9,14,15]. This is also supported by Lepawsky and McNabb [15], who argue that the Pollution Haven Hypothesis holds for e-waste trade; that is, a country with a lower Gross Domestic Product (GDP) per capita is more likely to be an importer of e-waste.

The identification of illegal e-waste smugglers is extremely difficult [16], thus, as trade is illegal, there are, by definition, no quantitative data available. However, the known and suspected routes of illegal e-waste trade routes are available [9]. With no data on e-waste net weights of shipments being available, further statistical analysis using the actual flows of e-waste trade is not possible. In order to examine the extent to which the sender and receiver countries differ in terms of economic and social development, we quantify their differences in scoring in terms of macroeconomic and social indicators for each known and suspected route. Up to this point, there has not been an example of similar research aiming at quantifying how socio-economic factors affect illegal e-waste trade. The outline of the rest of the paper is as follows: Section 2 covers the detailed research structure and the description of the selected indices, the results of the analysis are presented in Section 3 and discussed in Section 4, and Section 5 consists of the overall conclusions and further research suggestions.

## 2. Materials and Methods

In order to examine the relationship between socio-economic factors and known and suspected routes of illegal e-waste trade, we select two kinds of indices: macroeconomic and social. The two macroeconomic indicators are GDP per capita and the Open Markets Index (OMI), while the social indicators used in our analysis are the Human Development Index (HDI) and the Social Progress Index (SPI).

### 2.1. Gross Domestic Product Per Capita

GDP is the most commonly used indicator of a country’s economic evaluation, and is defined as “*the sum of gross value added by all resident producers in the economy plus any product taxes and minus any subsidies not included in the value of the products”* [17]. GDP per capita is a country’s GDP divided by its population. Data on GDP per capita (current USD) are obtained from the World Bank [17].

### 2.2. Open Markets Index

OMI, proposed by the International Chamber of Commerce (ICC) [18], is an index that measures the 75 selected countries’ openness to trade. It is calculated based on four weighed indicators (Trade Openness, Trade Policy, FDI Openness, and Trade Enabling Infrastructure). The examined countries’ scores range from 1 to 6, 6 being the highest.

### 2.3. Human Development Index

HDI [19] is an index calculated through the measurement of average achievements in three dimensions of human development: Long and Healthy Life (life expectancy), Knowledge (education), and A Decent Standard of Living (Gross National Income per capita). For each dimension, the geometric mean of the normalized indices is calculated to derive the HDI score for each country.

### 2.4. Social Progress Index

The SPI [20] is calculated based on three categories: Basic Human Needs, Foundations of Well Being, and Opportunity, with the overall measurement of “*12 components and 52 distinct indicators*”. It takes into account solely social and environmental indicators, excluding economic aspects, aiming at becoming a tool for policy makers to achieve higher social progress.

### 2.5. Scorings

In order to quantify the routes’ data, we record each route shown in the map in Lundgren’s [9] report, and divide the known and suspected routes. In known routes, the sender countries/regions are US, EU, South Korea, Australia, and Japan, and the receiver countries are Brazil, China, India, Mexico, Nigeria, Pakistan, Singapore, and Thailand. In suspected routes, the senders are the US and the EU, and the receiver countries are Argentina, Chile, Egypt, Haiti, Indonesia, Kenya, Malaysia, Philippines, Venezuela, Tanzania, United Arab Emirates, Vietnam, Russia, and Ukraine. For each index and each route, we assign the value “1” if the sender country has a higher GDP per capita or scores higher in HDI, OMI, or SPI than the receiver country, and “0” otherwise.

Following this, we calculate the percentage difference in each pair/route using the formula $\frac{y_{i}\;-\;\;x_{i}}{x_{i}}$ for the routes that are assigned the value “1”, and $\frac{y_{i}\;-\;\;x_{i}}{y_{i}}$ for the routes that are assigned the value “0”, with *x_i_* and *y_i_* denoting the respective route’s sender and receiver country’s score for each index.

## 3. Results

This section consists of the data on the four selected indices used in our analysis, the 0–1 classification, the percentage differences between the sender and receiver countries, their Z-tests and their visualization. The known and suspected routes are based on qualitative data by Lundgren’s report [9], using data from the University of Northampton of a global map with the known and suspected routes of e-waste. This map has also been employed in other authors’ analyses [21,22]. We have decomposed and repictured the aforementioned data, in order to separately approach the known and suspected routes of illegal e-waste trade. The results show that, with few exceptions, all sender countries have higher income per capita and score higher in OMI, HDI, and SPI than the receiver countries.

### 3.1. Known Routes of Illegal E-Waste Trade

**Figure 1:**
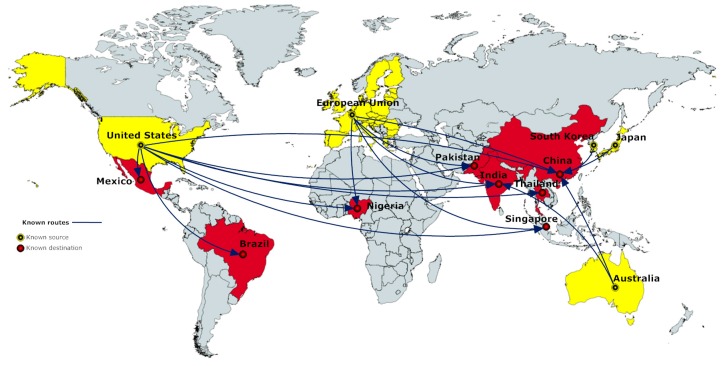
Known routes of illegal e-waste trade (Figure designed by the authors using data by the University of Northampton, not dated (n.d.), cited in Lungdren’s report [9]).

The known routes of illegal e-waste trade are shown in Figure 1. Note that the EU, the US, Australia, Japan, and South Korea are the countries that export to Mexico, Brazil, Nigeria, Pakistan, India, Thailand, Singapore, and China.

Table 1 and Table 2 consist of the data and the percentage differences in GDP per capita and OMI, and HDI and SPI, respectively, for the known routes of illegal e-waste trade. For each route, the value “1” is assigned if the sender country scores higher than the receiver country in the respective indicator, and “0” otherwise.

The average (STD) of the GDP per capita percentage differences between the sender and receiver countries in known routes is −75.62% (±36.93%), based on the data presented in Table 1. In the known routes of illegal e-waste trade, all receiver countries have lower GDP per capita than the sender ones, with the exception of Singapore; the only case where e-waste travels from lower income regions (US and EU) to a higher income one. For the second selected macroeconomic index, OMI, the picture is not different, as shown in Table 1. The percentage differences in the OMI scores in known routes have an average (STD) of −22.99% (±22.50%). As in the case of GDP per capita, the only exception is Singapore, which scores higher than the sender countries (US and EU).

The percentage differences’ average (STD) for the known routes in HDI is −24.02% (±13.77%). All routes except for EU to Singapore are from high to low HDI. The average (STD) for the known routes in SPI is −32.09% (±10.48%). For the SPI, no data for Singapore are available. For all other routes, we observe that e-waste trade occurs from a higher SPI-scoring country to a lower scoring one.

Overall, in all known routes for all four indices, we see that results are consistent; i.e., the sender country achieves higher scores than the receiver. The only exception for GDP per capita, OMI, and HDI is Singapore. The reason why Singapore (though it achieves higher scores than the sender countries in these indicators) is a receiver country could be attributed to the fact that it does not consider e-waste as hazardous and does not implement relevant legislations [9], thus encouraging illegal e-waste trade.

### 3.2. Suspected Routes of Ileegal E-Waste Trade

**Figure 2:**
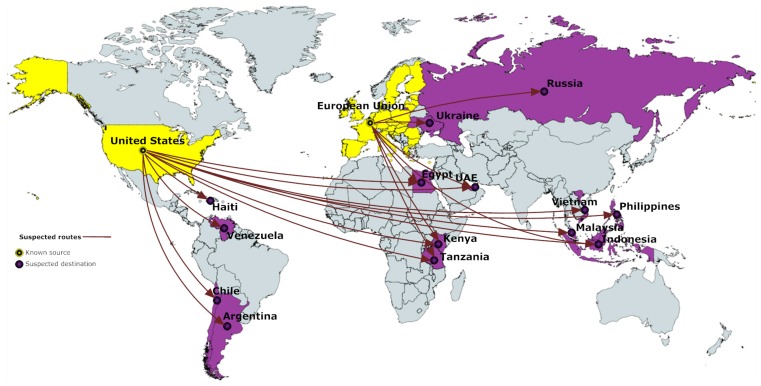
Suspected routes of illegal e-waste trade (Figure designed by the authors using data by the University of Northampton, n.d., cited in Lungdren’s report [9]).

The suspected routes of illegal e-waste trade are shown in Figure 2. The EU and the US are the only suspected exporters to Haiti, Venezuela, Chile, Argentina, Ukraine, Russia, Egypt, the United Arab Emirates, Kenya, Tanzania, Vietnam, Philippines, Malaysia, and Indonesia.

Table 3 and Table 4 consist of the data and the percentage differences in GDP per capita and OMI, and HDI and SPI for the suspected routes of e-waste trade.

In suspected routes, the results are consistent with those that are observed in known routes; i.e., e-waste travels from richer to poorer countries, with the only exceptions being from the EU to the United Arab Emirates (UAE) in GDP per capita, and from the US to Chile, Malaysia, and the UAE, and from the EU to the UAE in OMI. The average (STD) of the percentage differences are −79.83% (±30.83%) and −15.86% (±19.77%) for GDP per capita and OMI, respectively. This finding is consistent with previous studies [9,14,15] that have suggested that e-waste travels from economically developed to economically developing countries. For the HDI, the percentage difference average (STD) is −22.91% (±13.36), while for the SPI, the average is −23.38% (±11.10%).

Overall, for all four indices, we observe that the results for the suspected routes are consistent with those of the known routes; i.e., the sender countries score higher than the receiver ones.

## 4. Discussion

This section consists of the discussion of our results on the effect of macroeconomic and social factors on illegal e-waste trade, followed by the limitations of this research.

### 4.1. Illegal E-Waste Trade

As indicated in the results section, there is a strong connection between high scorings and sender countries in both known and suspected routes. Our results suggest that, for all indicators taken into account in the present study, illegal e-waste trade occurs from developed to developing countries, with the exceptions of Singapore in the known routes in terms of GDP per capita, OMI, and HDI (no data for the SPI), and in the UAE in the suspected routes in terms of GDP per capita, and in Chile, Malaysia, and the UAE in OMI, but not in HDI and SPI. This suggests that even though economic development in the UAE, for example, is higher, social development is not—thus supporting the hypothesis that illegal e-waste trade is not only a matter of economic evaluation, but further parameters need to be taken into account, such as social development, lack of or loose relevant legislation, and law enforcement efficiency.

Table 5 consists of the average percentage differences between sender and receiver countries in GDP per capita, OMI, HDI, and SPI, for both known and suspected routes of illegal e-waste trade. Though all differences are negative—i.e., the sender country scores higher in all indices in both types of routes—GDP per capita shows a highly increased difference between the sender and receiver countries, highlighting that even though income is not the only reason for illegal e-waste trade, it is the most significant one, at least in terms of what is examined in the present study.

**Figure 3:**
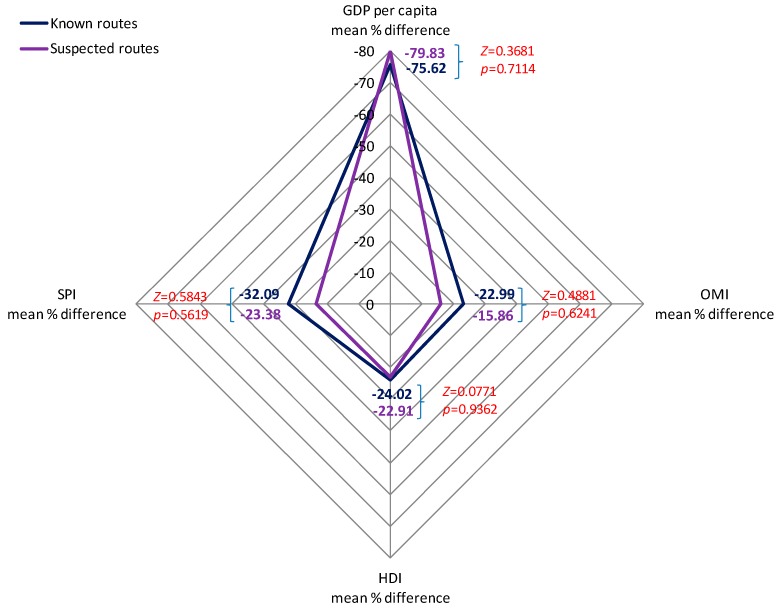
Average percentage differences in GDP per capita, OMI, HDI and SPI in known and suspected routes.

Figure 3 consists of the visualization of the average percentage differences between the sender and receiver countries in GDP per capita, OMI, HDI, and SPI for both known and suspected routes, in addition to the Z-tests of the percentages of each index in the two kinds of routes. We observe that the pattern between known and suspected routes is similar with small differentiations. In all four indices, the known and suspected routes’ differences are not statistically significant. So, basically, we observe that the known and suspected routes follow the same pattern in terms of differences in GDP per capita, OMI, HDI, and SPI.

Though illegal e-waste indeed flows from developed to developing countries, the issue of e-waste trafficking demands more complex approaches in order to be addressed, including international policy and regulations changes [12]. Law enforcement agencies worldwide need to lay some common ground regulations in order to prevent other types of crimes connected with environmental crime, such as tax evasion, drug trafficking [6], or money laundering [23]. Developing countries will continue to be the receivers of e-waste because of the lack of a specific regulatory framework addressing e-waste management.

This type of environmental crime will not cease to exist until the limits between legal and illegal e-waste trade are clearly defined. In order for this issue to be addressed, a universal definition of what constitutes e-waste needs to exist, in order to simplify the identification of e-waste flows. A common method of misleading the local authorities is the use of free trade zones (as is Batam Island in Indonesia, where international and national regulation is not applicable [16]), in an attempt to avoid any inspection or classification of e-waste by local authorities. In addition, the existence of a general code regarding e-waste would improve the detection of e-waste smuggling, as it would not permit the misclassification of products that are characterized as second-hand goods and are usually not taken into account in official statistics of e-waste trade. Furthermore, the existing codes are not harmonized, and they cannot be compared. Finally, the existing information on e-waste crime is based on controls made by the local authorities, which do not reflect the real extent of the crime [5,10]. These controls should be more intensive, as grey zones also exist [16], where little or no control takes place. For example, smugglers prefer to ship through Hong Kong, Taipei, and the Philippines in order to avoid detection from the Chinese enforcement officers, and then ship the containers to smaller ports in the mainland of China in order to let them reach their final destination [9].

### 4.2. Limitations

This study has some limitations. First, qualitative data are the basis of this research, and though the procedure of the quantification of the data and the obtained results are accurate, illegal e-waste trade cannot, by definition, provide quantitative data. Another data limitation is that the HDI does not take into account other aspects of human development, such as inequalities and poverty; thus, all social aspects are not fully integrated in the present research.

However, this study can provide ground for future research in illegal e-waste trade. Europe sends the majority of its e-waste to China [24] and West Africa (especially in Nigeria and Ghana [25]), while about 80% of the US’ e-waste is sent to developing countries, including China, Peru, Ghana, Nigeria, India, and Pakistan [24]. Though small-scale exports to West Africa do exist, the majority of e-waste flows to South–East Asia [9]. Despite the above, it is suggested that illegal e-waste trade occurs not only from developed to developing countries, but between developing countries as well, in addition to transboundary movements of e-waste between Asia and Africa [12]. Furthermore, e-waste that is sent to China––the country that receives the highest portion of e-waste on a global level [9]––is later exported to other countries in the region (e.g., Cambodia and Vietnam), while Singapore acts as a transit country, claiming e-waste as non-hazardous, thus not considering it to be under regulation [9]. The same holds for the UAE, which is the only country scoring higher in the GDP per capita and OMI than the sender country in suspected routes. Based on the above, further research should be done when more data on the changing of the routes are available, as the subject of e-waste trafficking between developing countries is not within the scope of this paper, and thus is not included in our analysis.

## 5. Conclusions

This paper aimed at quantifying macroeconomic and social aspects of illegal e-waste trade. As the only data on illegal e-waste trade are the known and suspected routes (i.e., the sender and receiver countries/regions), we examine the differences in GDP per capita, OMI, HDI, and SPI in relation to each respective route.

Our results show that both known and suspected illegal trade flows seem to occur from higher income to lower income countries, with the GDP per capita differences for the known routes having an overall of −75.62% average, and the suspected routes having an average of −79.83%. As this finding is also supported by the percentage differences in OMI, it is suggested that illegal e-waste is shipped from economically developed to economically developing countries, with notable differences. The same holds true for HDI and SPI, where—though with lower average percentage differences—it is observed that e-waste travels from socially developed to socially developing countries, with no exceptions.

Though many studies have already suggested that higher income countries illegally send e-waste to lower income ones (especially in the case of suspected illegal trade in e-waste), this paper highlights—for the first time in a quantitative way—the notable difference between the sender and receiver countries’ economic and social development. Further research should include the analysis of the illegal e-waste trade occurring between developing countries, countries that act as transit points of e-waste, and examine under what circumstances illegal e-waste trade takes place between developed countries (e.g., US to Hong Kong). In addition, an in-depth examination of such countries should be performed in the future, so as to determine the factors that make them attractive for such illegal activities.

The main issue in e-waste trade is that the limits between legal and illegal trade are not clear, thus this kind of environmental crime is bound to continue in the future. Nevertheless, in order to handle the illegal shipment of e-waste, what are necessary are (a) the implementation of strict and trade-specific worldwide legislative frameworks; (b) better controls by the local authorities; (c) a universal definition of what constitutes e-waste; and (d) a general harmonized code that would assist with the identification and detection of e-waste smuggling.

## Figures and Tables

**Table 1:** Illegal E-waste trade: percentage differences in GDP per capita and Open Markets Index (OMI) in known routes.

Countries		GDP per Capita				Countries		OMI			
Sender	Receiver	Sender	Receiver	Value	Diff.(%)	Sender	Receiver	Sender	Receiver	Value	Diff.(%)
USA	BRAZIL	54,629	11,384	1	−79.16	USA	BRAZIL	3.7	2.3	1	−37.84
USA	CHINA	54,629	7590	1	−86.11	USA	CHINA	3.7	3.0	1	−18.92
USA	INDIA	54,629	1582	1	−97.11	USA	INDIA	3.7	2.6	1	−29.73
USA	MEXICO	54,629	10,326	1	−81.10	USA	MEXICO	3.7	3.1	1	−16.22
USA	NIGERIA	54,629	3203	1	−94.14	USA	NIGERIA	3.7	2.4	1	−35.14
USA	PAKISTAN	54,629	1317	1	−97.59	USA	PAKISTAN	3.7	2.1	1	−43.24
USA	SINGAPORE	54,629	56,285	0	2.94	USA	SINGAPORE	3.7	5.5	0	32.73
USA	THAILAND	54,629	5977	1	−89.06	USA	THAILAND	3.7	3.5	1	−5.41
EU	CHINA	36,423	7590	1	−79.16	EU	CHINA	4.2	3.0	1	−28.57
EU	INDIA	36,423	1582	1	−95.66	EU	INDIA	4.2	2.6	1	−38.10
EU	NIGERIA	36,423	3203	1	−91.21	EU	NIGERIA	4.2	2.4	1	−42.86
EU	PAKISTAN	36,423	1317	1	−96.39	EU	PAKISTAN	4.2	2.1	1	−50.00
EU	SINGAPORE	36,423	56,285	0	35.29	EU	SINGAPORE	4.2	5.5	0	23.64
S. KOREA	CHINA	27,970	7590	1	−72.86	S. KOREA	CHINA	3.8	3.0	1	−21.05
AUSTRALIA	CHINA	61,925	7590	1	−87.74	AUSTRALIA	CHINA	4.1	3.0	1	−26.83
AUSTRALIA	INDIA	61,925	1582	1	−97.45	AUSTRALIA	INDIA	4.1	2.6	1	−36.59
JAPAN	CHINA	36,194	7590	1	−79.03	JAPAN	CHINA	3.6	3.0	1	−16.67

**Table 2:** Illegal e-waste trade: Percentage differences in Human Development Index (HDI) and Social Progress Index (SPI) in known routes.

Countries		HDI				Countries		SPI			
Sender	Receiver	Sender	Receiver	Value	Diff.(%)	Sender	Receiver	Sender	Receiver	Value	Diff.(%)
USA	BRAZIL	0.915	0.755	1	−17.45	USA	BRAZIL	82.850	70.890	1	−14.44
USA	CHINA	0.915	0.727	1	−20.49	USA	CHINA	82.850	59.070	1	−28.70
USA	INDIA	0.915	0.609	1	−33.47	USA	INDIA	82.850	53.060	1	−35.96
USA	MEXICO	0.915	0.756	1	−17.35	USA	MEXICO	82.850	67.500	1	−18.53
USA	NIGERIA	0.915	0.514	1	−43.82	USA	NIGERIA	82.850	43.310	1	−47.72
USA	PAKISTAN	0.915	0.538	1	−41.16	USA	PAKISTAN	82.850	45.660	1	−44.89
USA	SINGAPORE	0.915	0.912	1	−0.35	USA	SINGAPORE	82.850	-	-	-
USA	THAILAND	0.915	0.726	1	−20.67	USA	THAILAND	82.850	66.340	1	−19.93
EU	CHINA	0.866	0.727	1	−15.99	EU	CHINA	79.820	59.070	1	−26.00
EU	INDIA	0.866	0.609	1	−29.71	EU	INDIA	79.820	53.060	1	−33.53
EU	NIGERIA	0.866	0.514	1	−40.64	EU	NIGERIA	79.820	43.310	1	−45.74
EU	PAKISTAN	0.866	0.538	1	−37.83	EU	PAKISTAN	79.820	45.660	1	−42.80
EU	SINGAPORE	0.866	0.912	0	5.02	EU	SINGAPORE	79.820	-	-	-
S. KOREA	CHINA	0.898	0.727	1	−19.02	S. KOREA	CHINA	77.700	59.070	1	−23.98
AUSTRALIA	CHINA	0.935	0.727	1	−22.19	AUSTRALIA	CHINA	86.420	59.070	1	−31.65
AUSTRALIA	INDIA	0.935	0.609	1	−34.90	AUSTRALIA	INDIA	86.420	53.060	1	−38.60
JAPAN	CHINA	0.891	0.727	1	−18.31	JAPAN	CHINA	83.150	59.070	1	−28.96

**Table 3:** Illegal e-waste trade: Percentage differences in GDP per capita and OMI in suspected routes.

Countries		GDP per Capita				Countries		OMI			
Sender	Receiver	Sender	Receiver	Value	Diff.(%)	Sender	Receiver	Sender	Receiver	Value	Diff.(%)
USA	ARGENTINA	54,629	12,510	1	−77.10	USA	ARGENTINA	3.7	2.5	1	−32.43
USA	CHILE	54,629	14,528	1	−73.41	USA	CHILE	3.7	4.1	0	9.76
USA	EGYPT	54,629	3199	1	−94.14	USA	EGYPT	3.7	2.7	1	−27.03
USA	HAITI	54,629	824	1	−98.49	USA	HAITI	3.7	-	-	-
USA	INDONESIA	54,629	3492	1	−93.61	USA	INDONESIA	3.7	3.1	1	−16.22
USA	KENYA	54,629	1358	1	−97.51	USA	KENYA	3.7	2.4	1	−35.14
USA	MALAYSIA	54,629	11,307	1	−79.30	USA	MALAYSIA	3.7	4.0	0	7.50
USA	PHILIPPINES	54,629	2873	1	−94.74	USA	PHILIPPINES	3.7	2.9	1	−21.62
USA	VENEZUELA	54,629	-	1	-	USA	VENEZUELA	3.7	2.6	1	−29.73
USA	TANZANIA	54,629	955	1	−98.25	USA	TANZANIA	3.7	-	1	-
USA	UAE	54,629	43,963	1	−19.53	USA	UAE	3.7	4.7	0	21.28
USA	VIETNAM	54,629	2052	1	−96.24	USA	VIETNAM	3.7	3.6	1	−2.70
EU	EGYPT	36,423	3199	1	−91.22	EU	EGYPT	4.2	2.7	1	−35.71
EU	INDONESIA	36,423	3492	1	−90.41	EU	INDONESIA	4.2	3.1	1	−26.19
EU	KENYA	36,423	1358	1	−96.27	EU	KENYA	4.2	2.4	1	−42.86
EU	RUSSIA	36,423	12,736	1	−65.03	EU	RUSSIA	4.2	3.1	1	−26.19
EU	TANZANIA	36,423	955	1	−97.38	EU	TANZANIA	4.2	-	-	-
EU	UAE	36,423	43,963	0	17.15	EU	UAE	4.2	4.7	0	10.64
EU	UKRAINE	36,423	3082	1	−91.54	EU	UKRAINE	4.2	3.9	1	−7.14

**Table 4:** Illegal e-waste trade: Percentage differences in HDI and SPI in suspected routes.

Countries		HDI				Countries		SPI			
Sender	Receiver	Sender	Receiver	Value	Diff.(%)	Sender	Receiver	Sender	Receiver	Value	Diff.(%)
USA	ARGENTINA	0.915	0.836	1	−8.63	USA	ARGENTINA	82.850	73.080	1	−11.79
USA	CHILE	0.915	0.832	1	−9.07	USA	CHILE	82.850	78.290	1	−5.50
USA	EGYPT	0.915	0.690	1	−24.59	USA	EGYPT	82.850	59.910	1	−27.69
USA	HAITI	0.915	0.483	1	−47.21	USA	HAITI	82.850	-	-	-
USA	INDONESIA	0.915	0.684	1	−25.21	USA	INDONESIA	82.850	60.470	1	−27.01
USA	KENYA	0.915	0.548	1	−40.11	USA	KENYA	82.850	51.670	1	−37.63
USA	MALAYSIA	0.915	0.779	1	−14.83	USA	MALAYSIA	82.850	69.550	1	−16.05
USA	PHILIPPINES	0.915	0.668	1	−26.99	USA	PHILIPPINES	82.850	65.460	1	−20.99
USA	VENEZUELA	0.915	0.762	1	−16.72	USA	VENEZUELA	82.850	63.450	1	−23.42
USA	TANZANIA	0.915	0.521	1	−43.06	USA	TANZANIA	82.850	47.140	1	−43.10
USA	UAE	0.915	0.835	1	−8.69	USA	UAE	82.850	72.790	1	−12.14
USA	VIETNAM NAM	0.915	0.666	1	−27.21	USA	VIETNAM NAM	82.850	-	-	-
EU	EGYPT	0.866	0.690	1	−20.32	EU	EGYPT	79.820	59.910	1	−24.94
EU	INDONESIA	0.866	0.684	1	−20.99	EU	INDONESIA	79.820	60.470	1	−24.24
EU	KENYA	0.866	0.548	1	−36.72	EU	KENYA	79.820	51.670	1	−35.27
EU	RUSSIA	0.866	0.798	1	−7.87	EU	RUSSIA	79.820	63.640	1	−20.27
EU	TANZANIA	0.866	0.521	1	−39.84	EU	TANZANIA	79.820	47.140	1	−40.94
EU	UAE	0.866	0.835	1	−3.53	EU	UAE	79.820	72.790	1	−8.81
EU	UKRAINE	0.866	0.747	1	−13.74	EU	UKRAINE	79.820	65.690	1	−17.70

**Table 5:** Average percentage differences in known and suspected routes.

Index	Known Routes		Suspected Routes	
	Average % Difference	Standard Deviation	Average % Difference	Standard Deviation
GDP per capita	−75.62	36.93	−79.83	30.83
OMI	−22.99	22.5	−15.86	19.77
HDI	−24.02	13.77	−22.91	13.36
SPI	−32.09	10.48	−23.38	11.10

## Abbreviations

The following abbreviations are used in this manuscript:

GDP	Gross Domestic Product
HDI	Human Development Index
ICC	International Chamber of Commerce
OMI	Open Markets Index
SPI	Social Progress Index
UAE	United Arab Emirates
UNEP	United Nations Environment Programme
